# Quantifying Delivered Dose with Jet and Mesh Nebulizers during Spontaneous Breathing, Noninvasive Ventilation, and Mechanical Ventilation in a Simulated Pediatric Lung Model with Exhaled Humidity

**DOI:** 10.3390/pharmaceutics13081179

**Published:** 2021-07-30

**Authors:** Arzu Ari, James B. Fink

**Affiliations:** Department of Respiratory Care, College of Health Profession, Texas State University, Round Rock, TX 78665, USA; fink.jim@gmail.com

**Keywords:** aerosols, drug delivery, drug dosage, nebulizers, children

## Abstract

Acutely ill children may transition between spontaneous breathing (SB), noninvasive ventilation (NIV), and mechanical ventilation (MV), and commonly receive the same drug dosage with each type of ventilatory support and interface. This study aims to determine the aerosol deposition with jet (JN) and mesh nebulizers (MN) during SB, NIV, and MV using a pediatric lung model. Drug delivery with JN (Mistymax10) and MN (Aerogen Solo) was compared during SB, NIV, and MV using three different lung models set to simulate the same breathing parameters (Vt 250 mL, RR 20 bpm, I:E ratio 1:3). A heated humidifier was placed between the filter and test lung to simulate exhaled humidity (35 ± 2 °C, 100% RH) with all lung models. Albuterol sulfate (2.5 mg/3 mL) was delivered, and the drug deposited on an absolute filter was eluted and analyzed with spectrophotometry. Aerosol delivery with JN was not significantly different during MV, NIV, and SB (*p* = 0.075), while inhaled dose obtained with MN during MV was greater than NIV and SB (*p* = 0.001). The delivery efficiency of MN was up to 3-fold more than JN during MV (*p* = 0.008), NIV (*p* = 0.005), and SB (*p* = 0.009). Delivered dose with JN was similar during MV, NIV, and SB, although the delivery efficiency of MN differs with different modes of ventilation.

## 1. Introduction

The delivery of inhaled medications to children poses significant challenges due to their physiologic differences from adults. For instance, children have smaller airways, a shorter respiratory cycle, and lower tidal volume, vital capacity, and functional residual capacity [1,2]. Since children are nose breathers, upper airways filter inhaled gas. While aerosol deposition in children is lower compared to adults due to these physiologic differences, the reduced lung dose is body mass appropriate, and the weight-corrected doses can be similar to an adult’s [1,3,4]. More research is warranted to determine the correct dose, especially in infants and children.

Although a broad variety of aerosol devices and interfaces are on the market, they are designed for adults and require several steps to be used effectively. In addition, the lack of patient and parental education leads to ineffective delivery of aerosolized medications. Since most aerosol devices require multiple steps for optimum use, the correct delivery of aerosol therapy poses problems for children [5,6,7,8]. According to previous research, poor hand strength, inadequate breath holding and inappropriate inspiratory flow may be an issue with pressurized metered dose inhalers (pMDIs) [4]. Because an optimal inspiratory flow rate is essential for the correct use of dry powder inhalers (DPI) [9,10], children who are in acute conditions or younger than 6 years of age may not have the physical or cognitive abilities to achieve the required DPI flow rate. While nebulizers and pMDIs are commonly used in the treatment of children, they have different technical characteristics, and the selection of an aerosol device should be age-appropriate and individualized based on the patient’s psychomotor skills [11,12,13,14]. Furthermore, treatment and dose may vary from patient to patient depending on the mode of ventilatory support.

Although there are many publications on factors affecting aerosol drug delivery to children, no research has compared the delivery efficiency of pediatric aerosol devices in different modes of ventilation including mechanical ventilation, noninvasive ventilation, and spontaneous breathing. However, many children move between mechanical ventilation, noninvasive ventilation, and spontaneous breathing during their course of care. Regardless of the ventilatory support that they receive, the same drug dose was used. Furthermore, research on aerosol therapy in children mainly consists of in vitro studies that did not investigate the effect of exhaled humidity on aerosol drug delivery to this patient population. Although a 40% reduction was reported in the previous studies that used heated humidified ventilator circuits [15,16], they did not simulate active heating and humidity during expiration. Unlike the lung models used in previous research, children exhale heated and humidified gas that is close to body temperature. We found that using lung models without exhaled humidity overestimated aerosol deposition in simulated ventilator-dependent and simulated spontaneous-breathing tracheostomized adults. [17,18,19,20]. Unfortunately, no study has evaluated the effect of exhaled humidity on lung dose in children who receive different types of ventilatory support.

In a prior study, we simulated identical adult breathing parameters during aerosol administration with MV, NIV, and SB to compare inhaled dose distal to the trachea with both jet and mesh nebulisers [21]. This was the first study to show that aerosol delivery via MV with adult breathing parameters was greater than or equal to NIV and SB with both jet and mesh nebulisers, with similar delivery efficiency between NIV and SB. Based on these findings we were curious to explore how those findings might compare to similar protocol applied to pediatric models and breathing parameters. Therefore, the purpose of this study is to determine aerosol deposition with a jet nebulizer (JN), and mesh nebulizer (MN) during spontaneous breathing (SB), noninvasive ventilation (NIV), and mechanical ventilation (MV) using a pediatric lung model with exhaled humidity.

## 2. Materials and Methods

**Figure 1:**
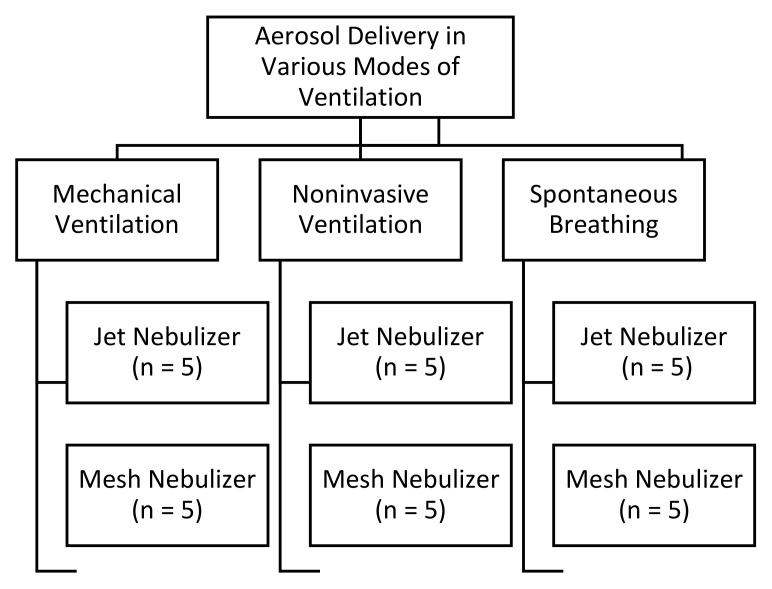
The plot of the study design with all aerosol devices and modes of ventilation tested in this study. Adapted from [21], ERJ Open Research, 2021.

An inhaled dose distal to the trachea was quantified during the delivery of aerosolized albuterol with a MN (Aerogen Solo, Aerogen Ltd., Galway, Ireland) and a JN (Mistymax10, Carefusion, Yorba Linda, CA, USA) during MV, NIV and SB using a similar pediatric breathing pattern and exhaled humidity (Figure 1).

### 2.1. Lung Models 

**Figure 2:**
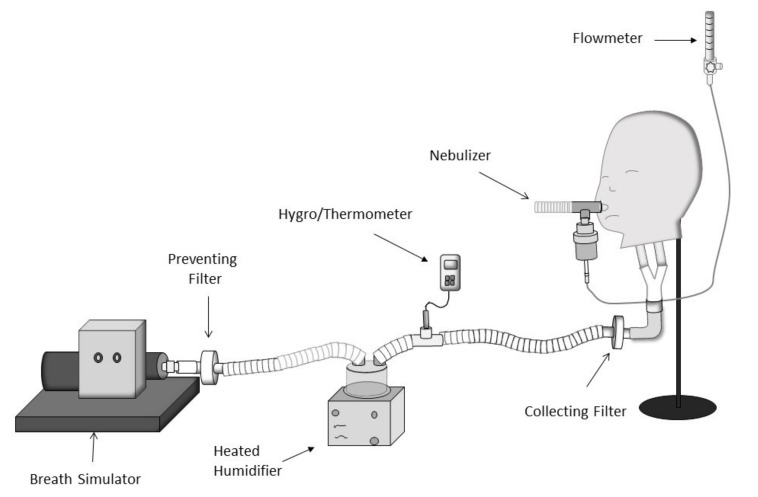
Lung model used to simulate a spontaneously breathing child. Adapted from [21], ERJ Open Research, 2021.

To simulate a spontaneously breathing child with a bodyweight of 31 kg, a teaching manikin was attached to a sinusoidal pump via a collecting filter (Respirgard II, Vital Signs, Totowa, NJ, USA) at the level of the bronchi and was connected to a breathing circuit via a heated humidifier (Fisher & Paykel Healthcare, Auckland, New Zealand) to simulate exhaled humidity (Figure 2). Breathing parameters were set at Vt 250 mL, RR 20 bpm, and I:E ratio 1:3.

A child receiving NIV was simulated through a ventilator (V60 Phillips Healthcare, Princeton, NJ, USA) with an un-heated single-limb circuit incorporating a fixed leak that was attached to a manikin via an oronasal facemask (AF541S, Respironics Inc., Murrysville, PA, USA).

**Figure 3:**
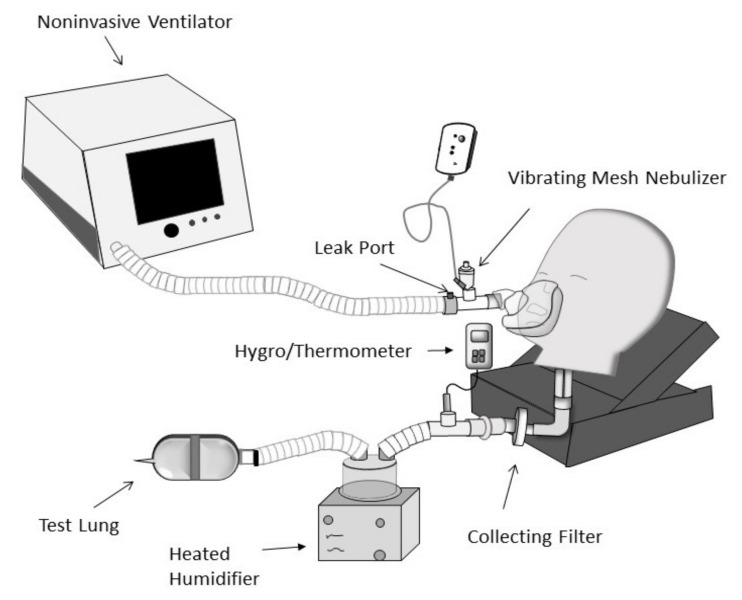
Lung model used to simulate a child receiving noninvasive ventilation Adapted from [21], ERJ Open Research, 2021.

Figure 3 shows the experimental lung model used to simulate a child receiving NIV in this study. A collecting filter placed distal to the bronchi of the manikin was attached to a test lung through a heated humidifier (Fisher & Paykel Healthcare, Auckland, New Zealand).

**Figure 4:**
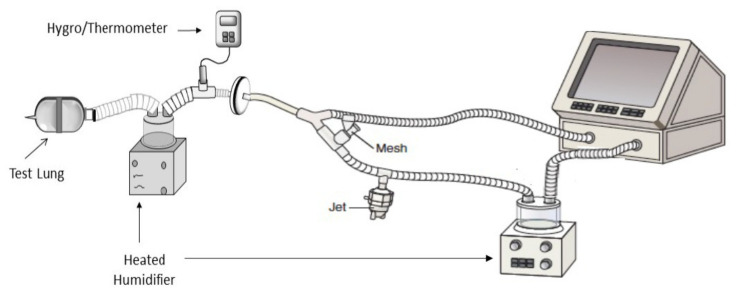
Lung model used to simulate pediatric mechanical ventilation. Adapted from [21], ERJ Open Research, 2021.

To simulate a mechanically ventilated child, a ventilator (Servo-i, Gettinge, Wayne, NJ, USA) was operated with a heated humidifier (Fisher & Paykel Healthcare, Auckland, New Zealand) with a heated-wire circuit attached to a 5 mm ID ETT (Figure 4).

The cuffed ETT was secured in a 15 mm ID/22 mmOD adapter and inserted into the inlet of a collecting filter (Respirgard II) with the tip of the ETT > 1 cm away from the filter media. Exhaled humidity (35 ± 2 °C, 100% relative humidity) was simulated with a heated humidifier (Fisher & Paykel Healthcare, Auckland, New Zealand) placed between the test lung and collecting filter.

Exhaled temperature and humidity were measured with a digital hygrometer/thermometer (Dwyer, Model 485, Michigan City, IN, USA) and the same breathing parameters (Vt 250 mL, RR 20 bpm, I:E ratio 1:3) were used in all lung models.

### 2.2. Data Collection 

Each nebulizer was placed at the Y piece of the inspiratory limb during MV, whereas they were positioned between the face mask and the leak port during NIV. A mouthpiece was used with each aerosol device during SB with the nares close, and the mouth of the manikin was sealed around the mouthpiece. Albuterol sulfate (2.5 mg/3 mL) was aerosolized with a JN and a MN until sputtering or the end of nebulization were observed, respectively. For SB, the JN was attached to a T-piece and 6-inch corrugated tubing, provided by the manufacturer. The MN was attached to a valved chamber (Aerogen Ultra, Aerogen Ltd., Galway, Ireland). Both the JN and MN were operated with supplementary oxygen of 6 L/min. The inhaled medication was collected in a filter at the level of the bronchi during each experiment. The collecting filters were capped and labeled at the end of each run for analysis.

### 2.3. Data Analysis 

Spectrophotometry (276 nm) was used to quantify the albuterol deposited on each collecting filter after elution with a 10 mL solution (20% ethanol with 0.1 N HCl). Means and standard deviations were calculated for total inhaled mass expressed as the fraction of the nominal dose added to each nebulizer during each experiment. Two-way factorial analysis of variance was used to analyze differences between inhaled doses obtained with the JN and MN during MV, NIV, and SB (*p* < 0.05).

## 3. Results

### 3.1. Impact of Different Types of Ventilation on Lung Dose

Table 1 shows the mean (± SD) percent of dose delivered with the JN and MN to the three pediatric lung models. Aerosol delivery with the JN was not significantly different during MV, NIV, or SB (*p* = 0.075), but aerosol deposition obtained with MN in a simulated ventilator-dependent child was significantly greater than NIV (*p* = 0.0001) and SB (*p* = 0.001). The delivery efficiency of MN during SB was significantly greater than for NIV (*p* = 0.01).

### 3.2. Impact of Nebulizer Type on Lung Dose

The delivery efficiency of the MN was better than the JN during MV (*p* = 0.008), NIV (*p* = 0.005), and SB (*p* = 0.009). Using the MN for aerosol drug delivery to simulated children increased the lung dose up to 3-fold compared to the JN, depending on the mode of ventilation used.

## 4. Discussion

This is the first comparison of aerosol administration during SB, NIV and MV with identical pediatric breathing parameters using a JN and MN. We found that the inhaled dose during MV was equal to or greater than NIV and SB.

Regardless of the type of nebulizer used, the inhaled dose efficiency trended lower with NIV than either MV or SB. Furthermore, this was the first study to simulate active heating and humidity during expiration in children.

### 4.1. Aerosol Delivery during Mechanical Ventilation

Our findings of aerosol deposition during pediatric mechanical ventilation are consistent with prior reports of Ari et al., in which dose efficiency distal to the ETT was 4.2% and 11.4% for the JN and MN, respectively, despite the difference in Vt (100 in the prior study vs. 250 mL) [16]. Previous research that evaluated how different tidal volumes affected aerosol drug delivery to children reported no difference in dose delivery efficiency comparing Vt volumes of 150, 200, and 300 mL [22]. Interestingly, a Vt of 100 mL delivered more drug than the larger volumes. This confirms speculation that during MV, with continuous aerosol generation, total inspiratory time per minute is a better predictor of inhaled dose. In other words, the greater proportion of aerosol-containing gas inhaled, the greater the lung dose. Other factors such as bias flow and inspiratory time also affected aerosol delivery during MV [16,23,24,25].

This may explain in part why Berlinski and Willis reported lower delivery efficiency of 2.8% and 8.7% with the JN and MN, respectively, while using a similar pediatric model with the nebulizers placed in a similar position 5.5 mm inner diameter cuffed ETT with parameters of Vt 200 mL, breathing frequency 20 breaths/min, PEEP 5 cm H_2_O, FiO_2_ 0.4, inspiratory time 0.75 s, inspiratory rise 0.15 s, flow trigger 3, bias flow 2 L/min, and heater 37 °C [26]. While reported deposition was lower, the relative differences between the JN and MN were consistent with prior reports.

### 4.2. Aerosol Delivery during Noninvasive Ventilation

Using a single-limb circuit with the JN and MN in a simulated spontaneously breathing 5-year-old child receiving NIV, Berlinski et al. reported an inhaled dose of 14.9% with the MN, and 5.5% with the JN at the IPAP/EPAP settings of 20/5 cm H_2_O [27]. When a similar model was used with a dual-limb NIV circuit and closed mask, the impact of the nebulizer type and position were more similar to the reports of standard mechanical ventilation than those of a single limb circuit [28]. This was likely due to the high flows generated by the turbine flow generator with a fixed orifice exhalation port with the single-limb circuit, which may have resulted in greater aerosol losses in the circuit and upper airways. This supports our findings of a lower inhaled dose with NIV than MV or SB in pediatric patients. Both in vitro and in vivo studies in adults, delivery dose efficiency with NIV using single circuits with turbine generators reported a lower inhaled dose with both the MN and JN than conventional mechanical ventilation with dual-limb circuits [29].

### 4.3. Aerosol Delivery during Spontaneous Breathing

Although research on aerosol drug delivery during pediatric MV and NIV is limited, several in vitro studies investigated aerosol deposition in simulated spontaneously breathing children. For instance, Restrepo and colleagues determined aerosol delivery with a JN attached to an aerosol mask in a spontaneously breathing pediatric lung model and reported a 2.88% delivery efficiency when there is no leak between the face and the mask [30]. Similarly, Cooper and Berlinski reported that, with a Vt of 300 mL, a nebulizer charge of 2.5 mg/3 mL deposited 73.6 µg of albuterol (2.9% inhaled dose) in a spontaneously breathing pediatric model using a JN with an aerosol mask [31]. The differences in our results can be explained by the interfaces used. For instance, we used a mouthpiece with the nebulizers and the others tested a JN with an aerosol mask.

In a study conducted by Lin et al. [32], the breathing simulator was set at a maximum muscle pressure of 13.5 cm H_2_O, a resistance of 20 cm H_2_O/L/s, and compliance of 5 mL/cm H_2_O, to generate an estimated tidal volume of 60 mL. The respiratory rate was set at 20 breaths/min; inspiratory time was 0.7 s; I:E ratio was 1:3; and inspiratory flow was 120 mL/s. Inhaled dose ranged from 2.18% with the standard mask, 2.65% with the dragon mask, and 3.67% with the fish mask. In this study, the Vt of 60 mL may have reduced the amount of aerosol reaching the trachea of the model. In a subsequent study, Lin et al. [33] modeled a 2-year-old child with a tidal volume of 150 mL, inspiratory time 0.8 s, peak inspiratory flow 20 L/min, and respiratory rate 25 breaths/min. The face and anatomical upper airway of a cardiopulmonary resuscitation manikin, representing the child, was modified to attach a bacteria filter to collect inhaled aerosol particles distal to the trachea. Using a standard face mask with nebulizers in this study, they reported an inhaled dose of 6.7% and 5.7% with the Neb-easy and LC Plus JN, respectively. Their findings showed that the long inspiratory time and a high respiratory rate would result in a higher inhaled dose than for the models with a lower inhaled minute volume.

In our previous research [34], we used a JN or MN with a spacer (Aerogen Ultra) to deliver albuterol sulfate (2.5 mg/3 mL) to a simulated spontaneously breathing pediatric lung model. (Vt 150 mL, RR 25 bpm, I:E ratio 1:2). The results of our study showed that aerosol deposition obtained from a JN plus the dragon mask and standard aerosol mask were similar (4.7% vs. 4.1%, respectively), while drug delivery from the MN via a valved-mask was greater (11.1%) than the other nebulizer and mask combinations. The higher delivery efficiency obtained from the MN in our previous research can be explained by the differences in I:E ratio and lung models. While we used an I:E ratio of 1:2 in our previous research, the I:E ratio of the current study was 1:3 with a shorter inspiratory time, which led to lower aerosol deposition. Furthermore, unlike the lung model in our previous research, the current study used lung models that simulated exhaled humidity during spontaneous breathing because children exhale heated and humidified gas close to body temperature. Using lung models without exhaled humidity may overestimate aerosol delivery compared to lung models with exhaled humidity.

In 2015, Lin et al. evaluated different face masks and flow rates with a high flow humidity system in a pediatric lung model (25–30 kg) with the following breathing parameters: tidal volume 250 mL, inspiratory time 1.0 s, and breathing frequency 20 breaths/min [35]. The delivery efficiency of the MN at 3 L/min was 7.3% with the OxyKid mask and 8% with the Dragon Mask. Using the face mask instead of a mouthpiece, as well as adding the flow and position of the nebulizer at the humidifier, may have resulted in a marginally lower inhaled dose than our findings of 8.9%.

Using a simulated spontaneously breathing pediatric model with Vt 250 mL, RR 20 bpm, and I:E ratio 1:2, Ari [36] reported an inhaled dose efficiency of 11.26 and 5.76% with the MN and JN, respectively. The lower aerosol delivery in this study can be explained by the I:E ratio and different lung models. For instance, we used an I:E ratio of 1:3, whereas Ari used a ratio of 1:2. While a spontaneously breathing pediatric lung model with exhaled humidity was used in this study, Ari’s lung model did not simulate exhaled humidification.

One of the few models comparing inhaled dose efficiency of the MN with SB and MV was reported by Parker et al. [37] simulating a 20 kg, 5-year-old child with parameters of 20 bpm, inspiratory time 0.75 s, and Vt 140 mL for both SB and MV. Their findings showed that the same emitted dose of Treprostinil resulted in similar inhaled doses for SB and MV (14.2 μg and 16.1 μg, respectively).

The reported differences in values for inhaled doses between labs were in part due to the differences in parameters and models. This was the first study that directly compared aerosol delivery with the same model and ventilation parameters for MV, NIV, and SB. It is remarkable that MV delivered similar or greater aerosol to the lower airways than did SB or NIV with either nebulizer type. Consequently, the expectation of lower aerosol delivery via an ETT during MV is not supported by our findings. It appears that the choice of nebulizer, especially the residual drug volume remaining at end of dose, has a greater influence on aerosol deposition than any measured differences with these three modes of breathing. These insights need to be further evaluated in this patient population with in vivo studies of deposition, pharmacokinetics, and pharmacodynamics.

### 4.4. Aerosol Delivery with Pediatric vs. Adult Model

Our findings in this pediatric model have several points of similarities and contrast with our prior adult model [21]. In the adult model, the inhaled dose with the MN (17–23%) was 3-fold greater than the JN (6.1–6.8%), however, with the pediatric model and breathing parameters the difference between MN (11.9–8.9%) and JN (4.9–2.97%) was less than 2 fold. In adults with NIV and SB were similar with each type of nebulizer, while NIV was lower than MV and SB in the simulated child. These differences can be attributed to the differences in anatomical models, the interfaces used and the breathing parameters and are likely representative of differences between pediatric and adult subjects in vivo. These observations support the value of conducting similar aerosol delivery testing across age specific models to provide guidance for nominal doses to achieve desired doses to the lung across interfaces. Further in vivo and in vitro testing is required to confirm and refine these observations.

## 5. Conclusions

The delivered dose to the pediatric lung model with the JN was similar during MV, NIV, and SB, while the delivery efficiency of the MN differed with different modes of ventilation. The MN increased the lung dose up to 3-fold compared to the JN, depending on the ventilation mode.

## Figures and Tables

**Table 1:** Mean (±SD) percent dose delivered with jet and mesh nebulizers during mechanical ventilation, noninvasive ventilation, and spontaneous breathing in children.

Type of Nebulizers	Mechanical Ventilation (MV)	Noninvasive Ventilation (NIV)	Spontaneous Breathing (SB)	*p*-Value
**Jet Nebulizer (JN)**	4.30 ± 0.61	2.97 ± 0.64	4.54 ± 0.88	0.075
**Mesh Nebulizer (MN)**	11.82 ± 0.65	7.25 ± 0.20	8.94 ± 0.34	0.001
***p*-value**	0.008	0.005	0.009	

## Data Availability

The data is included in the manuscript.
